# Anti-Tumor Effect of Steamed *Codonopsis lanceolata* in H22 Tumor-Bearing Mice and Its Possible Mechanism

**DOI:** 10.3390/nu7105395

**Published:** 2015-09-25

**Authors:** Wei Li, Qi Xu, Yu-Fang He, Ying Liu, Shu-Bao Yang, Zi Wang, Jing Zhang, Li-Chun Zhao

**Affiliations:** 1 College of Chinese Medicinal Materials, Jilin Agricultural University, Changchun 130118, China; w.li@jlau.edu.cn (W.L.); xuqi@jlau.edu.cn (Q.X.); liuying@jlau.edu.cn (Y.L.); wangzi8020@126.com (Z.W.); 2 Jilin Academy of Chinese Medicine Sciences, Changchun 130012, China; hyf_1992@163.com; 3 College of Animal Science and Technology, Jilin Agricultural University, Changchun 130118, China; yangshubao1981@163.com; 4 College of Pharmacy, Guangxi University of Chinese Medicine, Nanning 530011, China

**Keywords:** steamed *Codonopsis lanceolata*, H22 tumor-bearing, Bax, Bcl-2, VEGF, LC/MS

## Abstract

Although previous studies confirmed that steaming and the fermentation process could significantly improve the cognitive-enhancement and neuroprotective effects of *Codonopsis lanceolata*, the anti-tumor efficacy of steamed *C*. *lanceolata* (SCL) and what mechanisms are involved remain largely unknown. The present study was designed to evaluate the anti-tumor effect *in vivo* of SCL in H22 tumor-bearing mice. The results clearly indicated that SCL could not only inhibit the tumor growth, but also prolong the survival time of H22 tumor-bearing mice. Besides, the serum levels of cytokines, such as interferon gamma (IFN-γ), tumor necrosis factor-α (TNF-α), interleukin-6 (IL-6) and interleukin-2 (IL-2), were enhanced by SCL administration. The observations of Hoechst 33258 staining demonstrated that SCL was able to induce tumor cell apoptosis. Finally, immunohistochemical analysis revealed that SCL treatment significantly increased Bax expression and decreased Bcl-2 and vascular endothelial growth factor (VEGF) expression of H22 tumor tissues in a dose-dependent manner. Moreover, LC/MS analysis of SCL indicated that it mainly contained lobetyolin and six saponins. Taken all together, the findings in the present study clearly demonstrated that SCL inhibited the H22 tumor growth *in vivo* at least partly via improving the immune functions, inducing apoptosis and inhibiting angiogenesis.

## 1. Introduction

Hepatocellular carcinoma (HCC) is a serious human cancer with a high incidence and mortality throughout the world [1]. Although surgical managements or non-surgical therapeutic modalities have been employed, HCC is rarely curative [2]. Chemotherapy is one of the important methods for the treatment of tumor, but many lines of evidence showed that the antitumor activities of many chemotherapeutic agents result in severe side effects [3,4]. Recently, natural medicines with better effectiveness and lower toxicity have received more and more attention as a potential origin of new therapeutic anti-tumor drugs for HCC patients [5,6]. 

*Codonopsis lanceolata* (family Campanulaceae), a traditional medical plant, has been used in Asian countries for several inflammatory diseases, including asthma, tonsillitis, pharyngitis [7,8] and bronchitis [9,10]. Chemical analyses of this herb have revealed that the bioactive constituents are saponins, polyphenols, tannins, triterpene, alkaloids and steroids [11]. It has been reported that *C*. *lanceolata* had a protective effect against ischemic damage and alcoholic fatty liver [12]. In addition, *C*. *lanceolata* exerted better antioxidant, anti-obesity [13], antimicrobial and anti-inflammatory effects [14,15]. 

There is growing evidence that the heat-processing method could affect the chemical profile of herbals and lead to changes of the bioactivities. Among these heat-processing methods, steaming treatment is one of the most effective methods for Chinese medicines [16,17]. Taking steamed ginseng (red ginseng) as an example, a steaming process could cause extensive conversion of ginsenosides in unsteamed ginseng (white ginseng) to new less polar degradation compounds. Red ginseng is widely known to contain more pharmacological activities than white ginseng [18,19]. 

Prior to this investigation, several studies confirmed that steaming and the fermentation process significantly improved the cognitive-enhancement and neuroprotective effects of *C. lanceolata* [7,20,21]. In addition, recent studies have indicated that the extract of *C. lanceolata* exerted inhibition activities on HSC-2 (Hematopoietic Stem Cell-2) human oral cancer cells and HT-29 human colon cancer cell [22,23]. However, the anti-tumor efficacy *in vivo* of steamed *C*. *lanceolata* (SCL) still remains unknown. In the present study, to the best of our knowledge, the antitumor activity of SCL in an H22 tumor-bearing mice model was demonstrated for the first time. The possible molecular mechanisms by which the SCL performs its anti-tumor actions were investigated. 

## 2. Experimental Section 

### 2.1. Chemicals and Reagents

The roots of *C*. *lanceolata*, three years old, were collected in Jilin Province (Jilin, China). The specimens were identified and authenticated by Dr. Wei Li, Jilin Agricultural University. A voucher specimen (No. 20140311) has been deposited in the herbarium of the same college. Cyclophosphamide (CTX) was provided by Jiangsu Hengrui Pharmaceutical Co., Ltd. Hematoxylin and eosin (H&E) kits were acquired from Nanjing Jiancheng Bioengineering Research Institute (Nanjing, China). The Hoechst 33258 dye kit was obtained from Shanghai Beyotime Co., Ltd. (Shanghai, China). ELISA kits for mouse, including tumor necrosis factor-α (TNF-α), interferon-γ (IFN-γ), interleukin-2 (IL-2), interleukin-6 (IL-6) and vascular endothelial growth factor (VEGF), were purchased from R&D Systems (Minneapolis, USA). Rabbit monoclonal anti-Bax, anti-Bcl-2 and anti-VEGF antibodies were purchased from Cell Signaling Technology (Danvers, MA, USA). Other chemicals were all of analytical grade from Beijing Chemical Factory. 

### 2.2. Animals and Tumor Cell Lines

Mouse H22 hepatocarcinoma cells (H22) were obtained from Institute of Biochemistry and Cell Biology, Shanghai Institutes for Biological Sciences, Chinese Academy of Sciences (Shanghai, China), and maintained in the peritoneal cavity of male ICR mice, as previously described [24]. 

Male ICR mice, 22–25 g, were purchased from the Experimental Animal Holding of Jilin University with Certificate of Quality No. SCXK (JI) 2011-0004 (Jilin, China). Animals were housed individually in cages in a temperature-controlled room with a 12-hour light/dark cycle. After one week of acclimation with free access to regular rodent chow and water, the mice were used for further experiments. The experiments were conducted according to the Guide for the Care and Use of Laboratory Animals (Ministry of Science and Technology of China, 2006). All of the animal experimental procedures were approved by the Ethical Committee for Laboratory Animals of Jilin Agricultural University on 15 June 2013 (Approval NO. JLAU-ECLA-20140822).

### 2.3. Sample Preparation

The fresh roots of *C*. *lanceolata* were steamed in an autoclave at 105 °C for 1 h and then dried at room temperature (25 °C) to obtain steamed *C*. *lanceolata* (SCL). The SCL powders were extracted with 10 volumes of methanol using ultrasound-assisted extraction at a temperature of 40 °C for 90 min. After extracting 3 times, the obtained filtrate was concentrated under reduced pressure in a rotary evaporator to give a crude extract and to make up the solution for the animal experiment.

### 2.4. Animal Treatment and Experimental Design 

**Figure 1:**
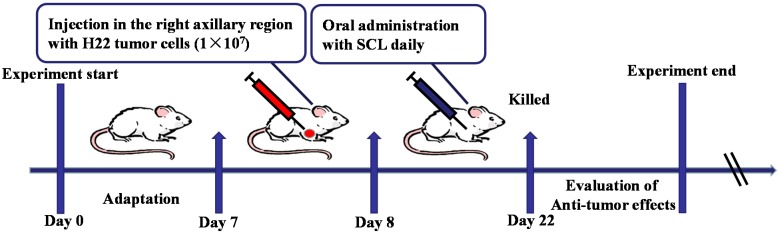
Experiment design scheme. To establish the murine solid tumor H22 transplanted model, mice were subcutaneously injected with H22 tumor cells. Twenty four hours after tumor cell injection, two doses of steamed *C*. *lanceolata* (SCL) (200 and 400 mg/kg) were administered daily for 14 days. At the end of the experiment, all mice were sacrificed to evaluate the anti-tumor efficacy. *n* = 10.

After an acclimatization period of one week, to establish the murine solid tumor H22 transplanted model, corresponding ascites tumor cells (0.2 mL, 2 × 10^6^ cells per mouse) were subcutaneously injected into the right axillary region of the mice in all groups (Day 7). Twenty four hours after inoculation (Day 8), the animals were randomly divided into five groups (*n* = 10) and treated for 14 days as follows: (i) the model group: animals received saline intragastrically; (ii) the CTX group (positive control group): animals received CTX (25 mg/kg) by intraperitoneal injection; (iii) the low and high dosage SCL-treated groups: animals received SCL test solution (200 and 400 mg/kg) intragastrically. The mice weights were recorded before and after each drug administration. Twenty four hours after the last administration of tested drug on the 22nd day of the experiment, blood samples were collected from the mice’s eyes, and serum was harvested by centrifugation. Then, all of the mice were sacrificed, and the whole bodies, the segregated tumor, thymus and spleen of the mice were weighed immediately. The tumor inhibitory rate was calculated by the following formula: tumor inhibitory rate % = (tumor weight of model group − tumor weight of tested group)/tumor weight of model group × 100. The volume of the solid tumor was measured with a digital caliper every other day. The values obtained were calculated according to the equation: *V* (mm^3^) = *A* × *B*^2^/2, where *A* and *B* represent the largest diameter and the smallest diameter, respectively. The tumor tissues of mice were collected for pathological sectioning with H&E and Hoechst 33258 staining. The experimental design is shown in Figure 1. 

### 2.5. Survival Assay

To measure the effect of SCL on survival time, thirty male ICR mice were inoculated with H22 tumor cells prepared by intraperitoneal inoculation, so as to observe the mice for a longer time period. The treatment was performed for 40 days, and the survival time of animals was monitored and recorded daily. The test continued for 40 days, and those that lived more than 40 days were defaulted as 40 days. The percent survival (%) was calculated using the following equation: percent survival (%) = ((10 − number of mice died in each group)/10) × 100.

### 2.6. Assay of Cytokines

A specific two-sided ELISA assay was performed to quantify serum levels of TNF-α, IFN-γ, IL-2, IL-6 and VEGF according to the manufacturer’s instructions. The absorbance was measured at 450 nm in an ELISA reader (Bio-Rad, CA, USA).

### 2.7. Hoechst 33258 Staining

Hoechst 33258 staining was performed as previously described with some modifications [25]. Briefly, at the end of the experiments, the mice were euthanized, and the transplanted tumors were dissected out and fixed in 10% neutral buffered formalin solution. We randomly chose three tumors from each group. Then, these samples were cut into 5-μm sections and stained by Hoechst 33258 (10 μg/mL). After being washed by PBS three times, stained nuclei were visualized under UV excitation and photographed under a fluorescent microscope (Olympus BX-60, Tokyo, Japan).

To quantify the fragmented and condensed staining, which indicated an apoptotic nucleus in the slides, we randomly chose five regions from the pictures of each tumor. These pictures were blinded and counted by two people, and their mean values were used in the statistical analysis. To avoid interobserver differences, a datum is valid only if the discrepancy between these two observers is less than 10%.

### 2.8. Histological Analysis of Tumor Sections

At the end of the experiments, H22 transplanted tumor tissues were fixed in 10% neutral buffered formalin solution. The washed tumor tissues were dehydrated in descending grades of ethanol and cleared in xylene, then embedded in paraffin. Sections were cut at a 5-μm thickness and stained with hematoxylin and eosin (H&E), then subsequently examined using a light microscope for histopathological examination.

### 2.9. Immunohistochemistry

Immunohistochemical analysis was performed as previously described [26]. Briefly, the 5 μm-thick paraffin sections were deparaffinized and rehydrated with a series of xylene and aqueous alcohol solutions, respectively. After antigen retrieval in citrate buffer solution (0.01 M, pH 6.0) for 20 min, the slides were washed three times with TBS (0.01 M, pH 7.4) and incubated with 1% bovine serum albumin for 1 h. The blocking serum was tapped off, and the sections were incubated in a humidified chamber at 4 °C overnight with primary antibodies against Bax (1:400), Bcl-2 (1:400) and VEGF (1:200), followed by a secondary antibody for 30 min. Substrate was added to the sections for 30 min followed by DAB staining and hematoxylin counter-staining. The positive staining was determined mainly by a brownish-yellow color in the nucleus of the cells. The immunostaining intensity was analyzed by light microscopy (Olympus BX-60, Tokyo, Japan). The immunohistochemical signal was assessed by estimating the area of the objects and the medium pixel intensity per object, as the optical density (OD). The Bax/Bcl-2 ratio is the optical density ratio of the Bax and Bcl-2 proteins.

### 2.10. LC/MS Analysis

Samples were analyzed on an Agilent HPLC system. Separation was achieved on a Hypersil ODS2 column (4.6 mm × 250 mm, 5 μm) from Dalian Elite Analytical Instruments Co. Ltd. The column temperature was set at 30 °C, and the detection wavelength was set 210 nm. The mobile phase consisted of acetonitrile (A) and water (B) with a flow rate of 1.0 mL/min. The gradient elution was programmed as follows: 0–20 min, 11%–16% A; 20–35 min, 16%–28% A; 35–60 min, 28%–60% A.

The HPLC-UV system was interfaced with the MS detector. The pneumatic-assisted electrospray positive ionization (ESI^+^) detection and cracking voltage is 160 V; the atomizing air pressure is 276 kPa (40 psi); the drying temperature is 350 °C; and the drying gas flow rate is 12 L/min.

### 2.11. Statistical Analysis

Statistical analysis was performed using SPSS 16.0. (Chicago, IL, USA). All values were expressed as the means ± standard deviation (SD). The differences between experimental groups were compared by analysis of variance (ANOVA) followed by a *t*-test. Differences with *p* < 0.05 were considered statistically significant. Drawings and survival curves were created using GraghPad Prism 6.04. Image-Pro plus 6.0 was used to quantify immunohistochemical analysis and Hoechst 33258 staining. 

## 3. Results 

### 3.1. Effect of SCL on Tumor Growth in H22 Tumor-Bearing Mice 

**Figure 2:**
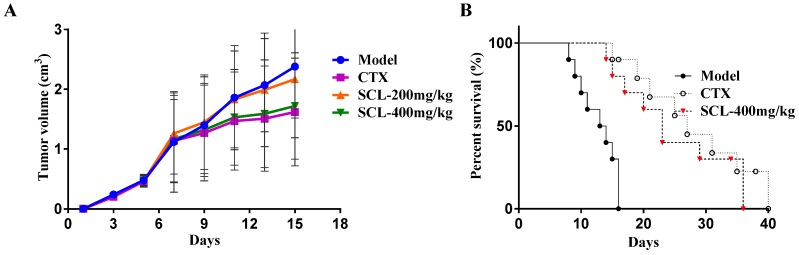
The effects of steamed *C*. *lanceolata* (SCL) on tumor growth (**A**) and life extension in H22 tumor-bearing mice (**B**). *n* = 10.

The antitumor effect of SCL on H22 tumor-bearing mice is summarized in Table 1 and Figure 2A–B. At the end of the study, the average tumor weight in the CTX group was significantly decreased (*p* < 0.01) compared to the model group. After treatment for 14 days, SCL with 200 and 400 mg/kg prior to the model group caused a significant decrease (*p* < 0.01). Accordingly, the tumor inhibitory rates of the CTX and SCL-treated groups were 81.73%, 46.08% and 60.87%, respectively. 

**Table 1:** Effects of steamed *C*. *lanceolata* (SCL) on tumor weights and relative organ index in H22 tumor-bearing mice. CTX, cyclophosphamide.

Groups	Dosage (mg/kg)	Weight (g)			Organ index (%)		Tumor weight (g)	Inhibitory rate (%)
		Before	After		Thymus	Spleen		
model	-	27.14 ± 1.43		33.96 ± 1.51	0.24 ± 0.11	0.69 ± 0.27	1.15 ± 0.85	-
CTX	25	27.17 ± 1.41		34.03 ± 0.69	0.13 ± 0.10 *	0.41 ± 0.07 **	0.21 ± 0.12 **	81.73
SCL	200	27.34 ± 1.34		33.70 ± 2.84	0.20 ± 0.08	0.63 ± 0.15	0.62 ± 0.47 **	46.08
	400	26.71 ± 1.05		32.23 ± 2.12	0.22 ± 0.05	0.66 ± 0.08	0.45 ± 0.19 **	60.87

Values are expressed as the mean ± SD, *n* = 10. * *p* < 0.05, ** *p* < 0.05 *vs.* the model group.

Figure 2A shows the tumor volume growth curves; the results indicated that the tumor volumes of the mice in the model group increased rapidly during the 14-day duration with their mean volumes reaching more than 2.3 cm^3^ at Day 15. In contrast, the treatment of SCL and CTX significantly suppressed the tumor growth (*p* < 0.05). From the ninth day, the average tumor volume in SCL- and CTX-treated mice had increased relatively slowly.

### 3.2. Effect of SCL on Life Extension in H22 Tumor-Bearing Mice

Figure 2C shows the effect of SCL on life extension in H22 tumor-bearing mice. The results indicated that all of the mice in the model group died within 16 days owing to the significantly fast growth of the H22 transplanted tumor. After treatment with CTX and SCL, half of the mice survived for more than 20 days. The average survival time of ascites H22-bearing mice treated with CTX and SCL at a dosage of 400 mg/kg was 40 and 36 days, respectively. Interestingly, the effect of SCL with 400 mg/kg was almost comparable to that of CTX. The findings clearly demonstrated that SCL treatment greatly prolonged the survival period of H22 tumor-bearing mice. 

### 3.3. Effect of SCL on Body Weight and Organ Indices in Mice

As shown in Table 1, there was no significant difference among the mean initial body weights of all groups. To evaluate whether SCL administration resulted in any side effect on the immune system, we determined the thymus and spleen indices of the host animals at the end of the study. The results indicated that spleen and thymus indices in the CTX-treated mice were significantly lower than the model group (*p* < 0.05, *p* < 0.01), which accounted for the immunosuppressive side effect by CTX during the therapy. However, there was no significant difference between the model group and SCL-treated groups. 

### 3.4. Effect of SCL on the Level of Serum Cytokines and VEGF

**Figure 3:**
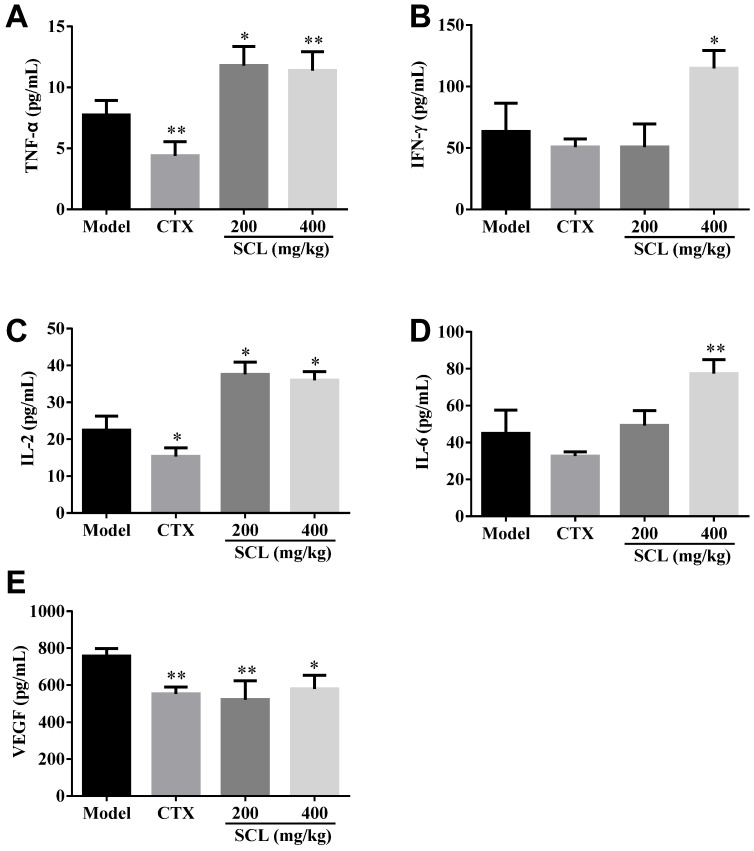
Effects of steamed *C*. *lanceolata* (SCL) on the levels of serum cytokines, including tumor necrosis factor-α (TNF-α) (**A**), interferon-γ (IFN-γ) (**B**), interleukin-2 (IL-2) (**C**), interleukin-6 (IL-6) (**D**) and vascular endothelial growth factor (VEGF) (**E**) in H22 tumor-bearing mice. All data were expressed as the mean ± standard deviation (SD), *n* = 10. * *p* < 0.05, ** *p* < 0.01 *vs.* the model group.

Since cytokines play a key role in controlling immune responses and inflammatory reactions, we employed the ELISA assay to investigate the effects of SCL on the production of serum cytokines, including TNF-α, IFN-γ, IL-2 and IL-6, in H22 tumor-bearing mice. As shown in Figure 3A–D, the serum levels of IL-2 and TNF-α were significantly increased in the SCL groups at doses of 200 and 400 mg/kg, respectively (*p* < 0.05). Mice treated with 400 mg/kg of SCL showed a significant increase in the serum levels of IL-6 and IFN-γ (*p* < 0.05). 

Angiogenesis, an essential process for tumor growth and metastasis, has become an important target for therapeutic intervention in many tumors. VEGF is recognized as a key contributor to the process of angiogenesis [27]. The serum level of VEGF was determined by an ELISA kit according to the manufacturer’s instruction. As shown in Figure 3E, the result indicated that SCL treatment at dosages of 200 and 400 mg/kg markedly decreased the serum level of VEGF (*p* < 0.05, *p* < 0.01), suggesting that SCL could repress angiogenesis in the H22 transplanted tumor.

### 3.5. Morphological Change by Treatment of SCL

**Figure 4:**
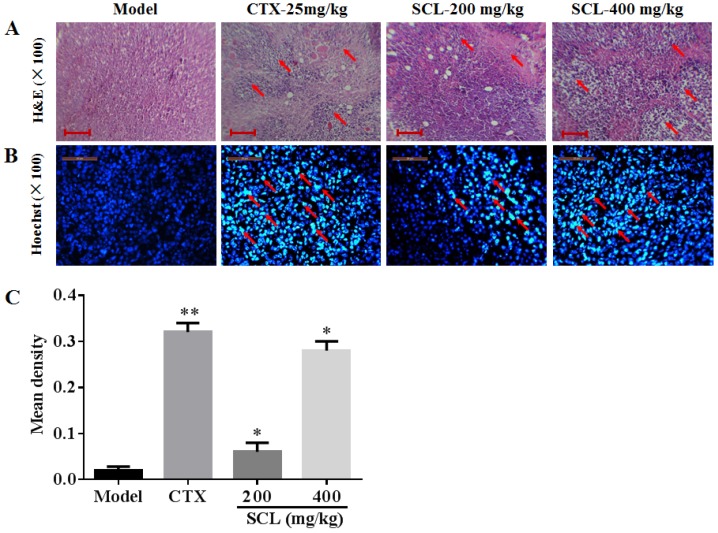
Histological examination of the morphological changes in tumors from H22-bearing mice. Tumor tissues stained with H&E (100×) (**A**) and Hoechst 33258 (100×) (**B**, **C**). The images were analyzed by an Image-Pro plus system. All data were expressed as the mean ± standard deviation (SD).* *p* < 0.05, ** *p* < 0.01 *vs.* the model group. The changes of the tumor cells are marked by arrow heads. Bar: 25 μm. *n* = 10.

In the present investigation, H&E sections of tumors were examined by microscopy. As shown in Figure 4A, tumor cells from the mice in model group were arranged tightly, having a large nucleus and a clearly apparent nucleolus. However, the tumor cells in SCL treatment groups exerted a loose arrangement and a large necrotic region. Furthermore, different increased degrees of vacuoles and vacuoles number were obviously observed in SCL-treated groups in a dose-dependent manner, which corroborates the significant anti-tumor efficacy of SCL on H22 tumor-bearing mice via inducing cell death.

To elucidate whether SCL treatment induced cell apoptosis in H22 transplanted tumors, Hoechst 33258 staining was performed. As depicted in Figure 4B, H22 tumor cells in the model group were observed as round-shaped nuclei with homogeneous fluorescence intensity, and most cell nuclei exhibited regular contours. After treatment with SCL and CTX for 14 days, significant nuclear fragmentation and condensation was observed in a dose-dependent manner (Figure 4C). 

### 3.6. Effects of SCL on the Expression of Apoptosis-Related Proteins

**Figure 5:**
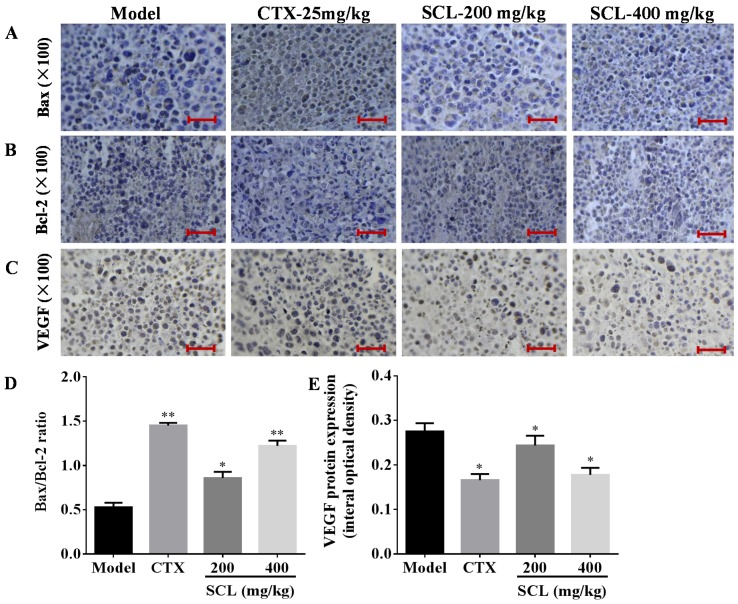
Effects of steamed *C. lanceolata* (SCL) on the expression of Bax, Bcl-2 and vascular endothelial growth factor (VEGF) (**A**–**E**). The protein expression was examined by immunohistochemistry. The images were analyzed by an Image-Pro plus system. All data were expressed as the mean ± standard deviation (SD). * *p* < 0.05, ** *p* < 0.01 *vs.* the model group. Bar: 25 μm. *n* = 10.

To find out the anti-tumor mechanism of SCL on H22 tumor-bearing mice, we examined the impact of SCL on the anti-apoptotic factor Bcl-2 and the pro-apoptotic factor Bax using immunohistochemical analysis. As shown in Figure 5A, B, high expression of Bcl-2 and low expression of Bax were observed in the tumor tissue sections of the model group. By contrast, SCL treatment decreased Bcl-2 expression and increased Bax expression of H22 tumor tissues in a dose-dependent manner. Interestingly, the ratio of Bax to Bcl-2, a rheostat of cell life or death, was increased in a dose-dependent manner after SCL treatment for 14 days. 

### 3.7. Effects of SCL on the Expression of VEGF

VEGF is considered an important growth factor implicated in tumor angiogenesis and can also be used as a tumor marker [27]. As shown Figure 5E, SCL treatment could significantly inhibit the expression of VEGF in a dose-dependent manner, coinciding with the decrease of VEGF level in serum. The above observation is a hint for the possible role of SCL as an angiogenesis inhibitor on H22 tumor-bearing mice. 

### 3.8. LC-MS/MS Analysis of SCL

**Figure 6:**
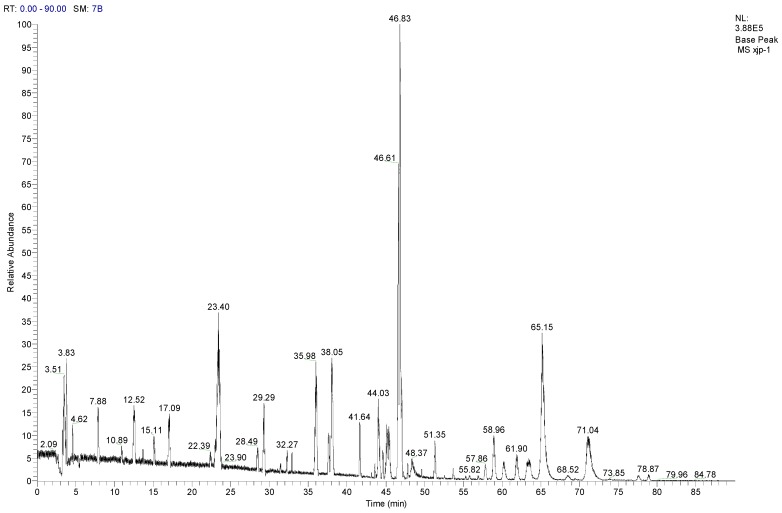
The total ion chromatogram of steamed *C. lanceolata* (SCL) (ESI^+^)

LC-MS/MS is considered a powerful tool for identifying and quantifying the compounds from natural medicines. As shown in Figure 6, in the present study, under the optimal chromatographic and MS conditions, one polyacetylene (lobetyolin) and six saponins were identified by comparing the retention time and by matching the empirical molecular formula with that of the reported known saponins [28,29,30]. The adduction of [M + NH_4_]^+^ would be generated when NH_4_OH was added to the mobile phase as a chromatographic modifier. All details of the compounds identified from SCL are summarized in Table 2. 

**Table 2:** Related substances of SCL identified by LC-MS/MS.

No.	Compounds	*t*_R_ (min)	Parent ion (*m*/*z*)	Formula	Ion type	Mr.	Product ion (*m*/*z*)
1	Tangshenoside I	23.4	696.9	C_29_H_42_O_18_	[M + NH_4_]^+^	678	193, 511, 513, 515, 678
2	Lobetyolin	28.49	414.2	C_20_H_28_O_8_	[M + NH_4_]^+^	396	295
3	Lancemaside F	43.61	1532.7	C_69_H_109_O_36_	[M + NH_4_]^+^	1514	267, 1371
4	Lancemaside B	44.14	1370.6	C_63_H_99_O_31_	[M + NH_4_]^+^	1352	-
5	Lancemaside G	44.21	1224.6	C_57_H_89_O_27_	[M + NH_4_]^+^	1206	-
6	Lancemaside A	45.14	1208.9	C_57_H_89_O_26_	[M + NH_4_]^+^	1190	-
7	Codonoposide I	47.85	1222.7	C_58_H_92_O_26_	[M + NH_4_]^+^	1204	-

## 4. Discussion 

HCC is one of the most common malignancies throughout the world with a high incidence and mortality [1]. Currently, though chemotherapy is one of the important treatment methods, the side effects and high toxicity limit its application [3,4]. Therefore, developing new therapeutic anti-tumor drugs with better effectiveness and lower toxicity has attracted growing interest. Many herbal medicines in China are found to be effective and to have relatively low toxicity, and they have attracted much attention with respect to treating tumors. Recently, *C. lanceolata* has been demonstrated to affect apoptosis and cell cycle arrest in colon cancer and leukemia cells [23,31]; therefore, we conjecture that it is likely to also have therapeutic potential against HCC. Furthermore, previous studies verified that steamed and fermented *C. lanceolata* possessed stronger biological and pharmacological activities [7,20,32]. Thus, the present investigation was designed to assess the anti-tumor effect *in vivo* of SCL on H22 tumor-bearing mice. 

In the present study, the *in vivo* anti-tumor efficacy of SCL was evaluated by the tumor growth inhibition and mice survival life prolongation rate of H22 tumor-bearing mice. A significant reduction of tumor volumes was observed in H22-bearing mice following SCL treatment at doses of 200 and 400 mg/kg in a dose-dependent manner. The survival time of tumor-bearing mice treated with the SCL was significantly longer than the model group. In addition, the results indicated that the spleen and thymus indices in all of the SCL groups were comparable to the model group, which clearly revealed that SCL administration could not result in any adverse effects on the immune system.

There is increasing evidence that cytokines regulate both cellular and humoral immune responses by affecting immune cell proliferation, differentiation and functions, which play a pivotal role in fighting against tumor growth [33]. TNF-α has been proven to be an effective anticancer agent in preclinical studies, by inducing apoptotic cell death and tumor necrosis [34,35]. IFN-γ is critical for the innate and adaptive immunity of bacterial and anti-tumor activities [36]. Recently, many studies have shown evidence that IL-2 and IFN-γ played an important role in specific immunological reactions to tumor cell growth, and they promoted innate and adaptive immune responses [37]. IL-6 is capable of inducing the proliferation of responsive T-cells. In this study, we investigated the effect of SCL on the production of cytokines, including TNF-α, IFN-γ, IL-2 and IL-6, in H22 tumor-bearing mice. The results showed that the serum levels of TNF-α, IFN-γ, IL-6 and IL-2 were significantly increased after SCL treatment compared to the model group (*p* < 0.05), which is in line with a previous report that *C*. *lanceolata* exhibited the ability to modulate macrophage-mediated immune responses [38]. To sum up, the finding indicated that the anti-tumor effect of SCL was achieved partly via increasing the immune response.

Generally, apoptosis has been characterized as a fundamental cellular activity to maintain the physiological balance of the organism [39]. Emerging evidence has demonstrated that the anticancer activities of certain chemotherapeutic agents are involved in the induction of apoptosis, which is regarded as the preferred way to manage cancer [40]. Previous studies have reported that TNF-α significantly increased the Bax genes, but significantly decreased the Bcl-2 gene level in in human tumor cells [34]. Interestingly, our results also showed that SCL could increase the level of TNF-α. In this study, apoptotic cells have been observed by Hoechst 33258 staining in the separation tumor section from H22 tumor-bearing mice [25]. The SCL treatment group induced cell apoptosis in the transplanted tumor, which further attested to the beneficial effects of SCL on H22 tumor-bearing mice. The mitochondria-dependent pathway was controlled by multiple layers of regulation, the most important players being members of Bcl-2 family [41]. There were two of the most important members regarding apoptosis in the Bcl-2 family, including the pro-apoptotic protein Bax and the anti-apoptotic protein Bcl-2 [42]. The increase in Bcl-2 expression caused resistance to chemotherapeutic drugs and radiation therapy, while the decrease in Bcl-2 expression may promote apoptotic responses to anticancer drugs [43]. Changes in the Bax/Bcl-2 ratio have been reported to be caused by downregulation of Bcl-2 and slight upregulation of Bax [44]. The findings from immunohistochemistry analyses of tumor tissues showed that the expression of Bcl-2 was significantly reduced, while the expression of Bax was relatively increased, which suggests that the SCL induced apoptosis by shifting the Bax/Bcl-2 ratio in favor of apoptosis. Interestingly, these results are in agreement with a previous study showing that *C*. *lanceolata* extract could induce apoptosis via increasing the Bax/Bcl-2 ratio and activation of caspase-3 in human colon tumor HT-29 cells [23]. We assumed that TNF-α also plays an important role in the process of SCL-induced apoptosis in H22 tumor cells, via regulation of the expression of Bcl-2 and Bax.

HCC is considered as a hypervascular tumor, and tumor growth and metastasis are angiogenesis dependent [45]. VEGF has an important effect on tumor angiogenesis usage in tumor therapy [27]. The present results showed that SCL significantly reduced the serum level of VEGF compared to the model group in H22 tumor-bearing mice. Furthermore, immunohistochemical analysis verified that SCL treatment could significantly inhibit the expression of VEGF in a dose-dependent manner, coinciding with the decrease of the VEGF level in serum. Recently, many lines of evidence have indicated that saponins from traditional Chinese medicines suppress tumor growth and angiogenesis in H22 tumor [46]. 

## 5. Conclusions 

In conclusion, our study proved that SCL exerted an anti-tumor effect in H22 tumor-bearing mice. The underlying mechanisms may be, at least in part, due to SLC improving the immune functions, inducing apoptosis and inhibiting angiogenesis. To the best of our knowledge, this is the first report to explore the anti-tumor efficacy of SCL on H22 tumor and the possible molecular mechanism involved, although further studies are needed prior to clinical use. 
